# Identification of the ubiquitin–proteasome pathway domain by hyperparameter optimization based on a 2D convolutional neural network

**DOI:** 10.3389/fgene.2022.851688

**Published:** 2022-07-22

**Authors:** Rahu Sikander, Muhammad Arif, Ali Ghulam, Apilak Worachartcheewan, Maha A. Thafar, Shabana Habib

**Affiliations:** ^1^ School of Computer Science and Technology, Xidian University, Xi’an, China; ^2^ Department of Community Medical Technology, Faculty of Medical Technology, Mahidol University, Bangkok, Thailand; ^3^ Computerization and Network Section, Sindh Agriculture University, Tando Jam, Pakistan; ^4^ Department of Computer Science, Collage of Computer and Information Technology, Taif University, Taif, Saudi Arabia; ^5^ Department of Information Technology, College of Computer, Qassim University, Buraydah, Saudi Arabia

**Keywords:** ubiquitin-proteasome pathway, DDE, protein sequence prediction, CNN, 2D-CNN

## Abstract

The major mechanism of proteolysis in the cytosol and nucleus is the ubiquitin–proteasome pathway (UPP). The highly controlled UPP has an effect on a wide range of cellular processes and substrates, and flaws in the system can lead to the pathogenesis of a number of serious human diseases. Knowledge about UPPs provide useful hints to understand the cellular process and drug discovery. The exponential growth in next-generation sequencing wet lab approaches have accelerated the accumulation of unannotated data in online databases, making the UPP characterization/analysis task more challenging. Thus, computational methods are used as an alternative for fast and accurate identification of UPPs. Aiming this, we develop a novel deep learning-based predictor named “2DCNN-UPP” for identifying UPPs with low error rate. In the proposed method, we used proposed algorithm with a two-dimensional convolutional neural network with dipeptide deviation features. To avoid the over fitting problem, genetic algorithm is employed to select the optimal features. Finally, the optimized attribute set are fed as input to the 2D-CNN learning engine for building the model. Empirical evidence or outcomes demonstrates that the proposed predictor achieved an overall accuracy and AUC (ROC) value using 10-fold cross validation test. Superior performance compared to other state-of-the art methods for discrimination the relations UPPs classification. Both on and independent test respectively was trained on 10-fold cross validation method and then evaluated through independent test. In the case where experimentally validated ubiquitination sites emerged, we must devise a proteomics-based predictor of ubiquitination. Meanwhile, we also evaluated the generalization power of our trained modal *via* independent test, and obtained remarkable performance in term of 0.862 accuracy, 0.921 sensitivity, 0.803 specificity 0.803, and 0.730 Matthews correlation coefficient (MCC) respectively. Four approaches were used in the sequences, and the physical properties were calculated combined. When used a 10-fold cross-validation, 2D-CNN-UPP obtained an AUC (ROC) value of 0.862 predicted score. We analyzed the relationship between UPP protein and non-UPP protein predicted score. Last but not least, this research could effectively analyze the large scale relationship between UPP proteins and non-UPP proteins in particular and other protein problems in general and our research work might improve computational biological research. Therefore, we could utilize the latest features in our model framework and Dipeptide Deviation from Expected Mean (DDE) -based protein structure features for the prediction of protein structure, functions, and different molecules, such as DNA and RNA.

## Introduction

Ubiquitination is used in many cellular processes, including signal transduction, cell division, and immune reactions. Many human diseases have been shown to be ubiquitinated, and recent progress in proteomic analysis has developed further interest in finding ubiquitination factors. Some proteins (eukaryotes) become ubiquitinated when they bind to a target protein residue (K), and this process controls cellular activities, such as signaling, cell division, and immunity. A number of important human diseases are associated with ubiquitination, and proteomic analysis has increased our interest in finding ubiquitination sites. However, small-scale data and shallow machine learning algorithms have been used in targeting location prediction software. The significant abundance of UCH-L1 in the brain and its presence in Lewy bodies and the role of UCH-L1 in the ubiquitin pathway suggest that it may be involved in Parkinson’s disease (Leroy et al., 1998). Based on the N-end pathway, the ubiquitination of synthetic substrates is efficient when the lines of the substrate rung protein are anchored at specific distances to the N-terminal ring (Suzuki and Varshavsky, 1999) (Zheng et al., 2000). Ubiquitin is found in most eukaryotic cells. Polypeptide hormones have been found in the calf thymus. The ubiquitin sequence is constant in all different organisms, such as cows, toads, and insects. Ubiquitin one may reflect important cellular properties that have changed slightly during evolution because of its extreme conservation and intracellular proteolysis. For these systems, ubiquinone is bound to a protein. The degradation of a protein *via* the ubiquitin–proteasome pathway (UPP, first) is initiated by the amino-terminal covalent attachment of multiple ubiquitin molecules (linkage), which involves two discrete and sequential steps: 26S proteasome (cleavage), followed by the final degradation of the complex that consists of the 19S proteasome, which has the 20S regulator as a constituent (degradation). Many immunologists believe that ubiquitin plays a role in cleaning up protein, regulating protein turnover, and creating new antigenic peptides (polypeptides) (Hershko et al., 1986; Hochestrasser et al., 1998). In addition, ubiquitin performs non-degradative processes, including DNA repair and endocytic regulation. The number of ubiquitin units attached to proteins determines whether a process follows a traditional or nontraditional pathway.

In 1975, Goldstein et al. (1975) first found the 3-step process of ubiquitination in higher eukaryotic organisms. Ubiquitin is fixed to lysine (K) residues. Three enzymes are involved in ubiquitination, namely, ubiquitin-activating enzyme (E1), ubiquitinating enzyme (E2), and ubiquitin-ligating enzyme (E3) (Welchman et al., 2005; Herrmann et al., 2007). The ubiquitination system regulates many aspects of cellular function, such as the location of proteins, the processing of proteins, and the breakdown of proteins (Hurley et al., 2006; Nath and Shadan, 2009). In addition, it is a key regulator of several biological processes, including cell division, transcription, DNA repair, signaling, transport, viral release, and intra-cellular movement. Research shows that ubiquitination, cellular transformation, immune response, and inflammation are intricately related. Moreover, several disorders are associated with abnormal ubiquitination status, such as neurodegenerative and inflammatory disorders. Ubiquitylation can be considered as another post-translation covalent signal and modification similar to acetylation, glycosylation, methylation, and phosphorylation (Roos‐Mattjus and Sistonen, 2004). In addition to targeting proteins for degradation, ubiquitylation has several roles.

In particular, when the ubiquitin levels and their associations are equal, ubiquitin aids in protein transport, DNA repair, DNA sorting, viral budding, RNA processing, RNA polymerase enzyme recognition, and infection. Unstable proteins, which cause the dysregulation of various regulatory pathways and unavailability of ubiquitin proteas, are important in neurodegenerative diseases, particularly as a feature in several types of proteinuria. Thus, the breakdown of the ubiquitin protection pathway and the identification of proteins associated with signals for the degradation of certain substrates would lead to new therapeutic approaches to the treatment of diseases with an impaired pathway of ubiquitin proteasome (Doherty et al., 2002). The ratio of ubiquitin molecules and ubiquitin associations is important in different functions; thus, ubiquitin can also have varying impacts on DNA repair, protein sorting, and viral budding. In many diseases such as neurodegenerative conditions, including Parkinson’s and cancer, the cytosolic and extraoral antigen genetic proteins undergo accelerated degradation and regulation, which involve many pathological pathway breaks or abnormal stability, thereby interfering with several regulatory pathways. Therefore, the dissection of the ubiquitin proteasome and proteins required for the degradation of ubiquitin substrates will lead to the development of new therapeutic treatments for many disease conditions, except for these two ubiquitin-protease deficiencies.

Cellular elements activate, transfer, eliminate, or identify several ubiquitin numbers. Given this complexity, the path of ubiquitin is ideal for an approach to system biology (Fu et al., 2019). Expanding the protein functions occurs when the ubiquitin protein binds to (attaches to) a lysine (K) residue (also known as ubiquitin or K protein, a regulatory amino acid) and serves as a regulator for cell division, immune reaction terminators, and signal transduction in eukaryotes. To date, research has shown that ubiquitination plays a key role in numerous human diseases, and recent developments in proteomics have attracted the attention of medical professionals. Although considerable data and high-processing intensity were not used in target sites, these computing tools are created using simple machine learning techniques. Tung and Ho (Tung and Ho, 2008) have created a comprehensive phenotypic amino acid feature database known as ubiquitin prediction (Kawashima et al., 2008) using 31 physicochemical characteristics, which can be found in published amino acid features (Radivojac et al., 2010). The ubiquitin prediction random forest algorithm, which uses 586 sequence attributes as an input function vector, has been used by Radivojac et al. (2010) (Zhao et al., 2011). In the voting mechanism, Zhao et al. (2011) (Lee et al., 2011) has adopted a whole approach. Lee et al. (2011) has developed (Chen et al., 2011) ubiquitin Site that uses the efficient kernel for RBF (radial basis) to identify all-purpose sites. Chen et al. (2011) (Cai et al., 2012) has proposed a k-spaced amino acid pair composition (CKSAAP) ubiquitin Site predictor using the CKSAAP. The predictor used the nearest neighboring algorithm proposed by Cai et al. (2012) by integrating four different types of predictive variables. In predicting ubiquitin, Cai and Jiang (2016) have used multi-player learning algorithms.

The study of protein- Ubiquitin–Proteasome association has become a key aspect of disease ubiquitin prediction. The Ubiquitin–Proteasome has the basic intention of disease pathogenesis and characteristics. Our proposed method achieved high performance of Ubiquitin–Proteasome, and then we have compared these various deep learning classification models. In appliance to protein sequence and proteins annotation information, we also provide a robust 2D Convolutional Neural Network based predictive model features analysis. Measurable structure-activity classification model obtained by machine learning technology can predict pathway-specific protein interaction and new signaling peptides. In this paper we proposed computational method is developed for prediction of sequence-based Ubiquitin proteins function. We have used computational models as such dipeptide deviation from expected mean (DDE) used as feature extraction models. We are one of the first uses of the 2D Convolutional Neural Network (2D CNN) in sequence-based Ubiquitin specific protein function prediction was our original preprint research on this subject. We propose an ubiquitin specific protein domains (Ubiquitin) sequence encoding system based on 2D Convolutional Neural Network (2D CNN) named 2DCNN-UPP. In addition, the prediction of Ubiquitin -specific of very small numbers of training instances, which is a major problem in the field of automated protein function prediction, has been tackled by the implementation of a realistic data enhancement approach of automated functional predictions previously developed through machine testing. Almost all cellular processes are regulated by the UPP, and deep learning algorithms are difficult to optimize on hyperparameters (Table 1).

**TABLE 1:** Sample for this experimental data points collected UPP and non-UPP sequences.

	Collected	Non-redundancy	Cross-validation	Independent
UPP	500	500	250	100
Non-UPP	810	755	700	400

## Materials and methods

### Datasets source

The respiratory datasets from the NCBI were utilized as a biotechnology, using the search phrase “ubiquitin proteasome path,” and then UPP proteins were collected from the NCBI (Pruitt et al., 2007).

The ubiquitin pathway-specific protein domain sequence was described as positive, which was called “known ubiquitin sequence.” All samples were drawn randomly to avoid introducing bias in the training set, and they served as independent test sets. This study used UniProt and Swiss-Prot databases, but only proteins specifically involved in ubiquitin/ubiquitin pathways in human were selected. Similarity of UPPS were removed through CD-HIT web service (Huang et al., 2010), from which 500 UPPs received 250 relevant pathway-associated proteins; in step one, only after adding UPPs, the desired protein sets were obtained. In the second step, the list of 325 Uprelated proteins was downloaded, and then 810 non-UP proteins were added after removing similarity using CD-HIT.

### Feature mining for discovering ubiquitin-pathway protein association

The ubiquitin-pathway protein sequence features were submitted for content analysis. Based on the structure, the researchers in this study discovered the physical and evolutionary features of proteins. If the characteristics can be further divided into two distinct varieties, then they may be considered as dipeptide deviation from expected mean (DDE) (Saravanan and Gautham, 2015) extending from a two-dimensional matrix to a 20 × 20 matrix. However, a compact functionality set was sought by finding a random projected matrix. Thus, a new compressive functionality has been discovered.

**FIGURE 1:**
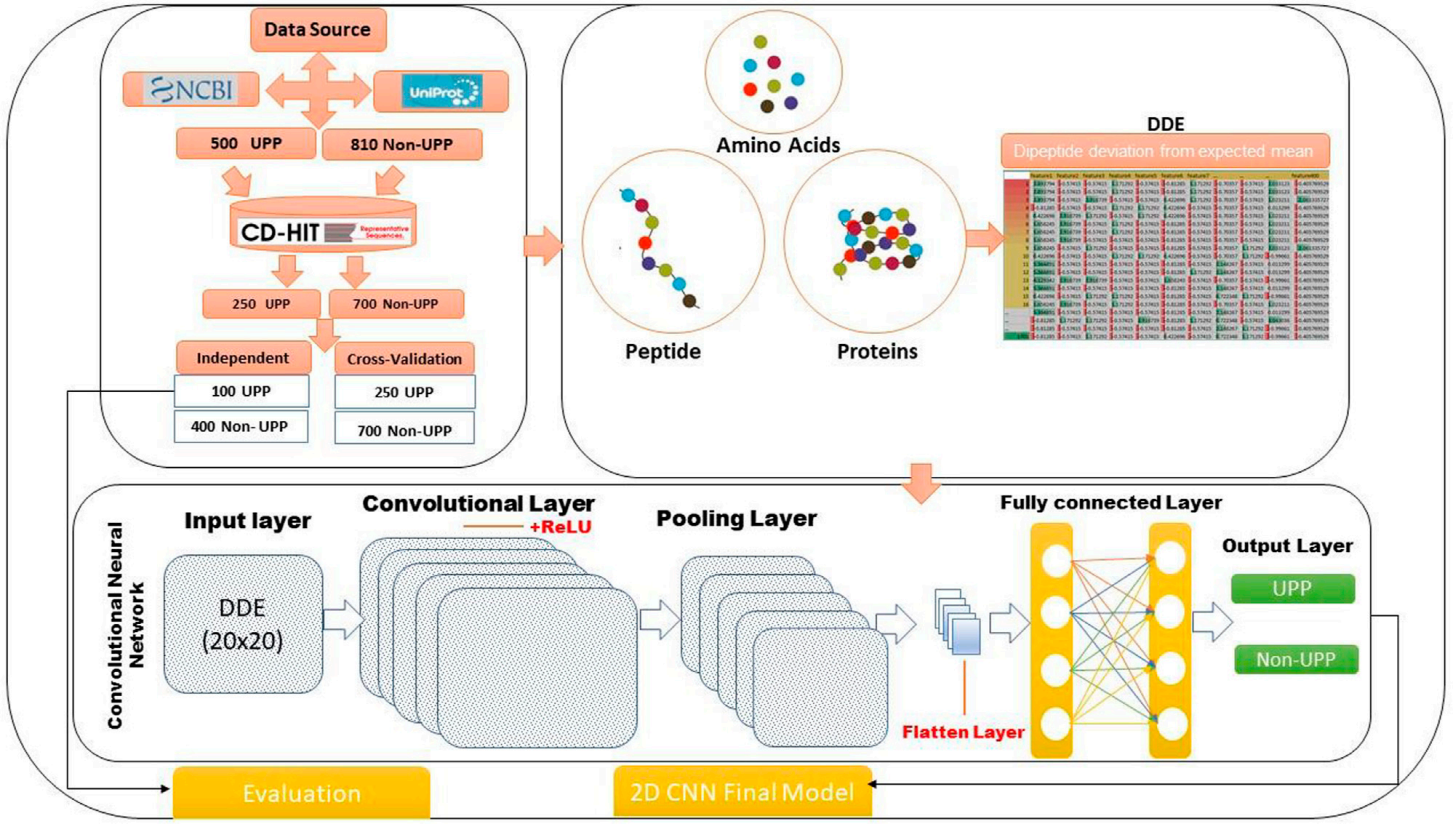
Method flowchart of the identification of UPP proteins using 2D-CNN.

The study focused on two-dimensional convolutional neural network (2D-CNN) and DDE, and an important method for classifying ubiquitin pathway proteins was developed. We conducted four analyses using experimental work: data collection, extraction of feature profiles, generation of 2D-CNN, and model evaluation. Figure 1 shows our flowchart of the method and gives the following details. This study consisted of 2D-CNN and UPP extraction feature profile matrices. Data on several variables were used, and an important technique for determining and organizing human pathways was developed. Physiochemical features were encoded using the DDE method for extraction vector profile matrix. A descriptor was based on evolutionary principles.

### Dipeptide deviation from expected mean

Feature formulation is one of the fundamental step in designing a computational model. How to capture the biological patterns from biological sequences is a challenging task. Thus, for this purpose feature descriptors are used for this purpose. The data were normalized by DDE and were treated to produce physicochemical features, features extracted from the ubiquitin pathway protein sequences, and information on evolution. The DDE, which has an all-amino-acid (making it easier to differentiate between nonspecific protein adsorption and nonspecific protein complexation) composition, was developed in this study. The ability of the DDE vector to increase the expression of specific proteins associated with ubiquitin pathway protection was verified, and its performance was then compared with various well-known pathways. DDE function vectors (also known as DDE-independent data or DDE feature extraction function for the vector matrix) had high predictive accuracy (accuracy demonstrated on a differential and independent data set).

The frequencies of amino acids were used for discovery (Saravanan and Gautham, 2015). A significant difference in mean values was found (task considered this difference as a feature), which might occur among their predicted median levels of acidity (Saravanan and Gautham, 2015). In this study, dipeptide composition characteristics were utilized to assess the number of deviations from the average di-predicted dipeptide numbers in accordance with earlier studies (Saravanan and Gautham, 2018). Means and standard deviations that define the DDE vector had a theoretical variance (***Tv***), second theoretical mean (***Tm***), and additional calculation through dipeptide composition (***Dc***). The research design was based on three computational calculations: ***D***_***C(i),***_ an indicator of ***Dc*** of the dipeptide of interest in peptide ***P***, which was estimated as follows:

$$D_{c\left(i\right)}=\frac{n_{i}}{N}$$ (1)

Numerous studies have investigated feature extraction (length samples) with 400 dipeptide feature properties (20 × 20). Length refers to the shape and properties of the samples, which have a dipeptide relationship, but the ones that were not useful were eliminated. However, ***I*** contend that dipeptide 1 and ***N*** are ***L-1***. Therefore, it is not equal to ***L-1*** (i.e., probable quantity in ***P***). *T*_*M(i)*_ indicates the theoretical mean.

$$T_{M\left(i\right)}=\frac{C_{i1}}{C_{N}}\times \frac{C_{i2}}{C_{N}}$$ (2)

In addition, the specified dipeptide ***C***_***i1***_ is the quantity of codons, and it is increased by the dipeptide ***C***_***i2***_ having the number of specified codons i. ***C***_***N***_ excluded the three stop codons, and the total number of codons in the amino acid sequence was known. ***T***_***M(i)-***_extracted peptides were not related to ***T***_***M(i***)_; hence, they were excluded. Therefore, 400 dipeptide features were retained, processed, and precomputed to generate compact dipeptide P. ***T***_***V(i)***_ theoretical variance was obtained by dipeptide and calculated as follows:

$$T_{v\left(i\right)}=\frac{T_{M\left(i\right)}\left(1-T_{M\left(i\right)}\right)}{N}$$ (3)

where i is the ***T***_***M(i)***_ statistical likelihood of expansion Eq. 2. The number of dipeptides in peptide P is the sum of ***N***, which is ***L-1***. ***DDE***_***(i)***_. Following its expansion, ***DDE(i)*** was presented as follows:

$$DDE_{\left(t\right)}=\frac{D_{c\left(i\right)}-T_{m\left(i\right)}}{\sqrt{T_{V\left(i\right)}}}$$ (4)

Finally, dipeptides were extracted to obtain the feature ***DDE*** of 400 dipeptides, and the extraction procedure used a 400-dimensional vector, which generated a collection of four 200 dimensional features.

$$DDE_{p}=\left\{DDE_{\left(i\right)},\ldots ,\ldots DDE_{\left(n\right)}\right\}\mathrm{,where,i=1,2,}\ldots \mathrm{,400}$$ (5)

### Two-dimensional convolutional neural network based framework

The present study used an approach that involved TensorFlow objects, which were different from matrix objects. The convolutional neural network (CNN) architecture is shown in Figure 1. Each CNN consisted of three thinned-out layers (the input included in a fully connected layers and pooling layer, including a convolutional layer), followed by hidden layers. CNN can be used in a wide range of areas, which can obtain remarkable results (Le et al., 2019a) and increase protein synthesis (Liu et al., 2016). As shown in Figure 1, a 2D-CNN UPP was utilized to illustrate the deep learning architecture. The TensorFlow backend was used in a custom library for our deep learning architecture, which was run on the Keras library with in python (Abadi et al., 2015; Mirabello and Wallner, 2018). In general, the 2D-CNN UPP was composed of multiple layers, each of which had a particular function to translate the input into a useful representation. After our 2D-CNN-architectured UPP was constructed, they were paired with an ordered architecture. Many previous studies in this field have proven that models can be optimized for certain architectures, and hyperparameters can be estimated by analyzing their predictive accuracy and predictive power. Furthermore, different levels of feature, hyperparameter, or parameters were required for numerous problems or datasets.

### Input layer for 2D convolutional neural networks

In this work, DDE features as a vector profile matrix matching the protein sequences were an input from the CNN (Ghualm et al., 2020). Therefore, we proposed a strategy for predicting UUP proteins using the DDE profile vector matrix as data input. The DDE profile vector matrix was presented as a grayscale image with 20 × 20 pixels. Then, we trained the model using the two-dimensional CNN system and this sort of data set. This is the first model to be used. We connected the input DDE profile vector matrix as Position-Specific Scoring Matrices (PSSM) to our 2D-CNN using a range of settings to improve model performance.

We aimed to capture as many hidden spatial characteristics as feasible in PSSM matrices by utilizing 2D CNN (Le and Nguyen, 2019). This methodology ensured the accuracy of the characteristics generated in the amino acid sequences and prevented disorder problem. The hidden layers were formed; the hidden features generated by CNN may easily identify UPP proteins. We used four filter layers (32, 64, 128, and 256) and three different kernel sizes in each filter for this method. When mentioning to convolutional neural networks, this filter is also sometimes referred to as a Windows or kernel. To create various feature mappings of the image, we can scan the image using a variety of filters. Each feature mapping will show the areas of the image that express the particular feature specified by our filter’s settings. Multiple filters make up each convolution layer. In reality, they take the form of a number like 32, 64, 128, 256, 512, etc. This is the same as a convolutional layer’s output channel count. In the independent data sets 32 filter size as sown the best performance. The same points were inserted into independent sets as an ubiquitin specific pathway protein family. The training performance was then evaluated using a 10-fold cross-validation. 2D-CNN image-based data points served as the input layer of a CNN. The images in the preceding discussion were presented using a two-dimensional matrix. Furthermore, we created an image with a dimension of 20 × 20 = 400 before entering the input. Having “m” input will allow for “m” forms of variation (400, m).

### Zero padding layer

The present study used a research design to investigate two categories of cushioning, including valid name and padding. Our convolutional layer was not padded, and our input size was not maintained if we provided true padding. Before we converted the original input into the size of the input, we padded our model. We can add columns and rows of zero values to the top, bottom, left, and right of the feature profile matrix. When a 20 × 20 input feature extracted matrix was used, the production frequency was 20 × 20 window per matrix. Our model did not have distinct output dimensions after the filters were applied to the input data. The zero padding 2D architecture incorporated the 2D CNN 20 × 20 zero matrices at the end of the chain. The shape of our network increased by 20 rows and 20 columns with the addition of zero padding. When we applied the effect to the input dimensions, the output was unchanged (Zhao et al., 2019).

### Convolutional layer

The target population was categorized on the basis of each 2D CNN analysis of the input multichannel 2D image convolution computation and extract features in its plane. Each convoluted kernel was concentrated in the width and height of 2D input volumes of the previous layer to calculate the points between kernel and entry. All the input volumes resulted in a 2-dimensional activation map for each kernel. The essential building block of a CNN was the convolutional layer. The parameters of the layer consisted of an array of filter learning (or kernel), which had a limited field, but such parameters stretched over the entire input volume depth (Yan et al., 2019; Cheng and Parhi, 2020).

### Activation layer

The activation provided by all forward layers can either be utilized through an activation layer or by applying the function of activation of the rectified linear unit, which would result in the typical activated ReLU value with default values: max (x, 0), the maximum element level of 0, and the tensor input. The default parameter modification allowed the utilization of non-zero thresholds, the max value of activation, and multiple non-zero input values below the threshold (Zhao et al., 2018).

f(x)=max(0,x) (6)

### Pooling layer

Each overall layer was followed by a max pooling layer, which selected and filtered features on the outcome of the overall layer to accomplish fluid compression in one degree. In general, a pooling layer is inserted in a CNN architecture periodically. The pooling layer could reduce the spatial dimension of the representation to decrease the number of computation parameters in the network and control the overlay. On each input depth section, the pooling layer functioned independently, and it could be resized by max pooling layer functioning. The most common form was a bundling layer with 20 × 20 filters, applied with 20 downsamples of every input depth slice by two along the width and height, with 75% of the activation being discarded (Yamashita et al., 2018).

### Dropout layer

Dropout is a technique of regularization to alleviate neural network overfitting. In particular, dropout discards information by randomly zeroing every hidden neural network node during the workout (Krizhevsky et al., 2012; Billones et al., 2016). Thus, the network can benefit from the combined effect of small subnetworks, achieving a good regularization effect. Unlike fully connected layers, leaving the convolutional characteristic map was not effective. The strong correlation between spatially adjacent pixels of the convolutional characteristic map and redundant textual information was shared. Therefore, the conventional pixel dropout cannot reject the information on the convolutional map entirely. Although dropout was frequently used to regularize deep neural networks, applying it to fully connected and convolutional layers was fundamentally different. Moreover, dropout was different from the deep learning community. Therefore, dropout function was only useful to fully connected layers with a value of 0.02.

### Flatten layer

Flat layers were the first few layers in the output layer. Considering that all classes required probability distribution in the output layers, flatten layers converted the input matrix to a vector. This output could be used to generate information in the following layers. Then, we observed a thick layer, which was a fully connected regular neural network. The classification of the features from the convolutional layers and grouping layers in this layer was carried out. The model will deactivate the neurons in a layer randomly with some probability p in the drop-out layer. The neural network ignored selected neurons in the course of training when the dropout value was added to a layer, ensuring a quicker training time. The dropouts of 0–1 were used in this study to evaluate our model. The ReLU played an important role as an activation function, which was used to classify motor superfamilies during the construction of the CNN (Le et al., 2019b).

### Fully connected layer

Neurons in a completely connected layer were fully connected, as shown by regular neural networks, to all activations in the previous layer. Therefore, their activations can be calculated by multiplying the matrix and then offsetting the bias. For additional information, see the Neural Network section of the notes (Ullah et al., 2021). The fully connected layer entered the concatenated vector of the combining layer, where the units in the previous layer were fully connected to the units. The combination of features was performed, and complex relationships in this layer were modelled. We used a hidden layer in our experiment.

### Loss function

Binary cross entropy function is given their potential to deal with class imbalance, the loss functions compared with this work have been selected. Log loss functions were analyzed in a binary classification (front and background), The actual class output, which can only be either 0 or 1, is compared to each of the projected probabilities using binary cross entropy. Binary jobs use the loss function known as binary cross-entropy. This function tasks that respond to an inquiry with just two options (e.g., yes or no, A or B, 0 or 1, and right or left). The negative average of the log of the corrected predicted probabilities used in classification issues is known as binary cross entropy or log loss. Imagine that any classification could only be reduced as a binary option (e.g., 0 or 1, A or B, and yes or no). The score that penalizes the probabilities based on how far they are from the predicted value is then calculated. Depending on how near or far the value is to the actual value. Cross entropy a loss function with the appropriate class mark as the goal and maximizing likelihood value. To avoid overfitting, a range of regularization techniques can be used (for example, protein 1 or protein 2 penalties, which are frequently used in suggested models), like adding penalties to the loss function. The loss function has been demonstrated to be effective for a variety of binary classification problems (Jia et al., 2018; Tran et al., 2019).

### SoftMax utilization

X is the neural network number of inputs. The model output was calculated by a SoftMax function that was used to calculate the probability of each output. The logistic function SoftMax was defined by the following formula. The output of the model was quantified using a SoftMax function, which minimized the probability of any output (Abdel-Hamid et al., 2013; Zhang et al., 2017). The model produced multiple real numbers, which represented the probability that the sample may be included in each classification category. For probability computation of each category, the SoftMax function as illustrated in Eq. 1 was used: Trainable parameters with 339,170 data points were established in the model (Table 2).

$$\sigma {\left(z\right)}_{i}=\frac{e^{z_{i}}}{\sum_{k=1}^{k}e^{z_{i}}}$$ (7)

**TABLE 2:** Trainable parameters used in the 2D CNN model.

Layer (type)	Output shape	Param #
zero_padding2d_35	(ZeroPaddi, none, 3, 10, 50)	0
conv2d_56 (Conv2D)	(None, 1, 8, 32)	14432
activation_29 (Activation)	(None, 1, 8, 32)	0
max_pooling2d_56	(MaxPooling, none, 1, 4, 16)	0
zero_padding2d_36	(ZeroPaddi, none, 3, 6, 16)	0
conv2d_57 (Conv2D)	(None, 1, 4, 64)	9280
activation_30 (Activation)	(None, 1, 4, 64)	0
max_pooling2d_57	(MaxPooling, none, 1, 2, 32)	0
flatten_21 (Flatten)	(None, 64)	0
dropout_30 (Dropout)	(None, 64)	0
dense_53 (Dense)	(None, 64)	4160
activation_31 (Activation)	(None, 64)	0
dense_54 (Dense)	(None, 2)	130
activation_32 (Activation)	(None, 2)	0

### Hyperparameters

Deep UPP agnostic indicated that loss function was strongly dependent on the hyperparameters. In this study, we described the individual layers and their hyperparameter and connectivity details. Hyperparameters were architecture-level parameters, which differed from the parameters of a model that has been trained to reproduce. When developing a deep learning model, the options of these hyperparameters were governed by a series of factors (Le et al., 2019c). The performance of the model had a significant impact. For example, the number of convolutional layers, number of filters in each layer, number of periods, dropout rates, and optimizers were important hyper parameters, which could affect the deep-learning model. In adjusting the hyperparameters, we should speed up the workout and avoid overfit. As suggested by Chollet (Bergstra et al., 2011; Hutter et al., 2011), the hyperparameter tuning process was followed by each step of the above-mentioned hyperparameter tuning approach.

### Performance evaluation of the model

Analysis was performed to determine whether a specific ubiquitin pathway protein sequence was a UPP protein or not; thus, UPP proteins were defined as “positive,” whereas non-UPP proteins were defined as “negative.” We described TP, FP, TN, and FN as true positive, false positive, true negative and false negative, respectively. In the formula no. 7–10, TP stands for correctly predicted UPP and TN for correctly predicted non-UPP, whereas FP stands for incorrectly predicted UPP proteins and FN for incorrectly predicted non-UPP. The assessment metrics were then defined in accordance with (Zhang et al., 2019). The current study primarily aimed to predict whether or not a sequence was a UPP protein; hence, we used the definition of the UPP protein as “positive” and non-UPP protein as “negative.” We trained the model for each dataset with a 10-fold cross-validation technique on the training dataset. Hyper-parameter optimization was used to find the best model for each dataset based on the 10-fold cross-validation results. Finally, the predictive ability of the current model was assessed using an independent dataset. The measurement methods used to measure the predictive performance of our model included sensitivity, specificity, accuracy, and MCC.

$$\mathrm{Specificity=}\frac{\mathrm{TN}}{\mathrm{TN}+\mathrm{FP}}$$ (8)

$$\mathrm{Accuracy}=\frac{\mathrm{TP}+\mathrm{TN}}{\mathrm{TP}+\mathrm{FP}+\mathrm{TN}+\mathrm{FN}}$$ (9)

$$\mathrm{Sensitivity}=\frac{\mathrm{TP}}{\mathrm{TP}+\mathrm{FN}}$$ (10)

$$MCC=\frac{TP*TN-FP*FN}{\sqrt{\left(TP+FP\right)\left(TP+FN\right)\left(TN+FP\right)\left(TN+FN\right)}}$$ (11)

### Protein-protein interaction (analysis of UPPs using PPIs)

STRING is a large database of known and predicted protein-protein signal interactions. The output is presented in the form of nodes and edges, respectively, representing interactions with the protein. Available evidence suggests that exit scores are an indicator of confidence, meaning that STRING is likely to evaluate interactions correctly. Instead, it is used to predict the possible interactions of multiple proteins. Several selected protein names were mentioned in the input and *Homo sapiens* was selected as the organism (Taju et al., 2018).

## Results

Result analysis consisted of quality and reliability of research modelling methods, which were major factors in the study. Initially, we conceived an experiment through analysis of data, calculation and comparison of results, and discussions. Primarily, an experiment was developed through the evaluation of data, calculation of results, and comparison of numerous consultations and results. Based on our research, we used two models, including the DDE model. An experimentally validated ubiquitination predictor was required for proteomic-scale proteomics. Using a convolutional network predictor, four processes had different approaches, each of which borrowed a portion of the sequences and physical properties. Cross-validation using 2DCNN-UPP had an AUC of 0.9, sensitivity of 100%, and specificity of 83%. The MCC reached zero point one and a half times higher or approximately 78% more thoroughness. Model cross-validation sets as training and independent sets as test validation were poorly understood, and the loss function and accuracy were measured with cross-entropy loss function.

### Ubiquitin-proteasome pathway and non-ubiquitin-proteasome pathway sequence for amino acid composition

The amino acid composition of UPP and non-UPP sequences was determined by calculating their frequency. By computing the frequency among these amino acids, we analyzed the composition of UPP and non-UPP. The amino acids in two different datasets contributed significantly to the highest frequency. The two types of data did not differ much, but some points were present. Amino acids C and P occurred at the highest frequencies around the proteins of UPP. On the contrary, amino acids L and I were produced at high frequencies around non-UPP proteins. These amino acids played a vital part in the identification of UPP proteins. Thus, through special features of these amino acid contributions, our models could accurately predict UPP proteins.

### Two-dimensional convolutional neural network was set to train the model

A total of 150 epochs were used as a model of “train” in our proposed model. A feature function returned an object, and then the object features were used in a history feature expansion to create loss and accuracy visualization, allowing a user to have control over the amount of information to see while evaluating between training and validation (White et al., 2017). Consequently, 2D-CNN has been trained and has remained equal to 0.8023, and the corresponding increase in time and the corresponding reduction in precision loss (significance) were only 0.7558. Overfitting can occur when the network interpreted the training data well but cannot distinguish between the outside world and the hidden world; thus, the quality of training and testing varied. Therefore, a dropout layer and anisotropic model were added to the network to examine the changes of these parameters as the first additional layers were removed. Finally, model’s efficiency was examined to determine whether obtaining a specific conclusion was possible.

### Test set model evaluation

**FIGURE 2:**
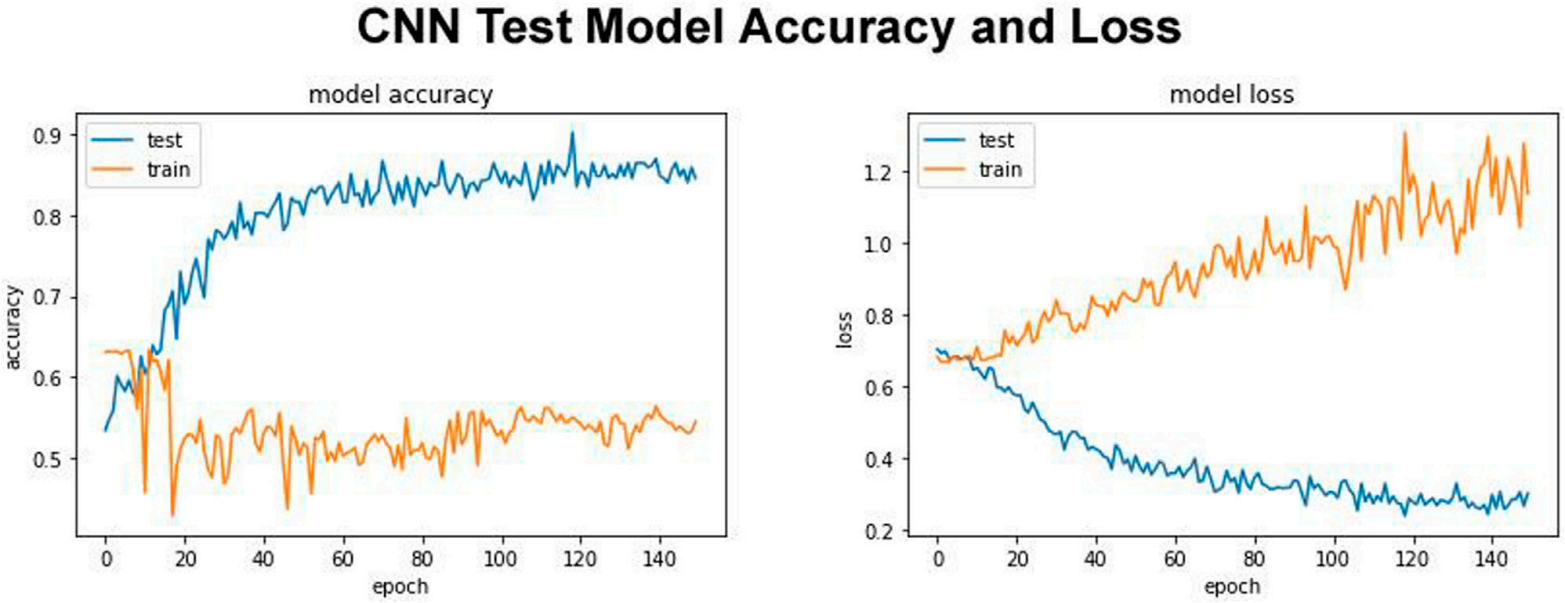
Test model accuracy and model loss.

Our proposed DDE model with test model accuracy score 0.8760 and test loss of 0.2044 is shown in Figure 2. The quality of the results of the test was remarkable. In contrast to more traditional models, this model also holds up with deep learning models (White et al., 2017). Our proposed model trained using the training data, and its fitness will be evaluated using the validation model. We modify the hyperparameters, such as the network’s number of layers, the number of nodes per layer, the number of epochs, etc. The training exercise at the parameter or hyperparameter level must not involve the Test Set. This model was used to evaluate, estimate, and plot the accuracy and bias from training to validation data (Figure 2). The expandable parameter was used to define the number of neuron fractions to drop for this step. We will only keep the active node, and the number of dormant nodes was reduced; therefore, no data was saved. Therefore, we did not explore, compile, and retrain the network, but no further expansion was performed in other regions. We work with a network with an input batch size of 10 and more than 150 epochs.

### Performance of the classification results for the identification of the ubiquitin-proteasome pathway

The earlier results indicated that the use of Tensor Flow backends and model building was in accordance with the findings (Rahmati et al., 2017). The two-dimensional CNN architecture was in production. Next, the maximum number of consecutive hidden layers was 32, followed by a top-down expansion with two separate convolutional layers, resulting in a depth of 64, 128, and 256. Table 3 summarizes the models created by the DDE using the cross-validation values for various filter numbers. An expansion strategy could identify true sequences with a 10-fold cross-validation with accuracy of up to 0.862 using independent sets, and then whereas an exhaustive validation method could obtain true sequences with a 10-fold cross-validation accuracy of 0.838. However, other filter numbers showed average results, and the results were higher compared with other studies. Our cross-validation set gave us a specificity of 0.832, indicating that the percentage of all examples were not excluded correctly, and MCC was only 0.680. The data produced consisted of a number of independent datasets utilizing various filter numbers. Using our independent and pairwise algorithm, we obtained a accuracy of 0.862 with regard to the target feature set, a mean corrected proportion of 0.921 sensitivity score, and MCC of 0.730. Therefore, we obtained our model structure and used it with the evolutionary structure of the environment. The final model that we built was Adadelta, a robust performance optimizer, which could expand the capacity by applying the following five hyper parameter models.

**TABLE 3:** Performance of the classification results for identification (UPP) using different filter layers.

Filter numbers	Cross-validation				Independent			
	Sens	Spec	Acc	MCC	Sens	Spec	Acc	MCC
32	0.844	0.832	0.838	0.680	0.921	0.803	0.862	0.730
32-64	0.846	0.810	0.828	0.659	0.763	0.735	0.750	0.506
32-64-128	0.846	0.791	0.818	0.640	0.696	0.731	0.714	0.429
32-64-128-256	0.842	0.816	0.829	0.662	0.739	0.687	0.714	0.432

**FIGURE 3:**
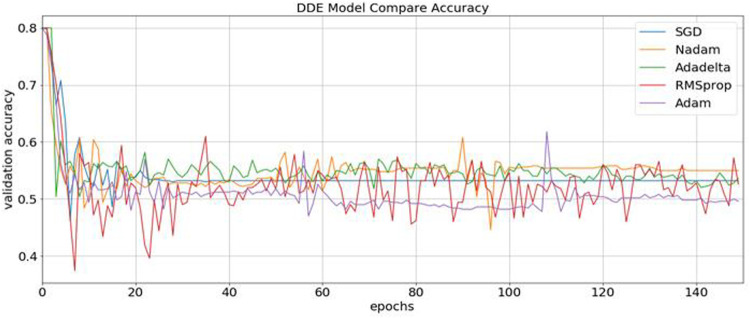
Identification of the validation accuracy of the ubiquitin protein pathway based on different optimizers ranging from 0 to 150.

Consequently, our model was built using the concatenation of these buried layers. Then, the neural networks were trained using three different methods, namely, RMSprop, Adam, and SGD, followed by an expansion phase, in which Nadam and Adadelta were applied. During each round of optimization, a new network was created to evenly match the individual optimizers with the old network before starting the process. Figure 3 shows that the information was collected and compared.

We finally arrived at a model, which was developed by making an option between an advanced algorithms (Adam) with a proven track record of consistent performance. The optimizer (algorithm) that was suitable for the proposed model was Adam. Similarly, during the experiment, the number of iterations was set (float, default = 0.001 steps). The batch size was fixed at 10, the dropout rate = 0.2, Kernel size = 3 was used as a tuning parameter, which permitted adjustments between 100 and 150, as shown in Table 4. In addition, we went through the model to validate its results, and then the results were tested to ensure their accuracy and established in the context of another using a separate set of data. Considering that the training algorithm was accurate at the 150th epoch, our model iteration expanded to include model validation results, thereby preventing our models from developing too much dependence on project’s dataset. Consequently, we accomplished at a short duration by redesigning our training to prevent overfitting.

**TABLE 4:** Optimal hyperparameters used in our proposed method.

Used hyperparameter	Values
Number of epochs	80
Learning rate	0.001
Batch size	10
Kernel size	3
Dropout rate	0.2
Optimizer	Adam

**FIGURE 4:**
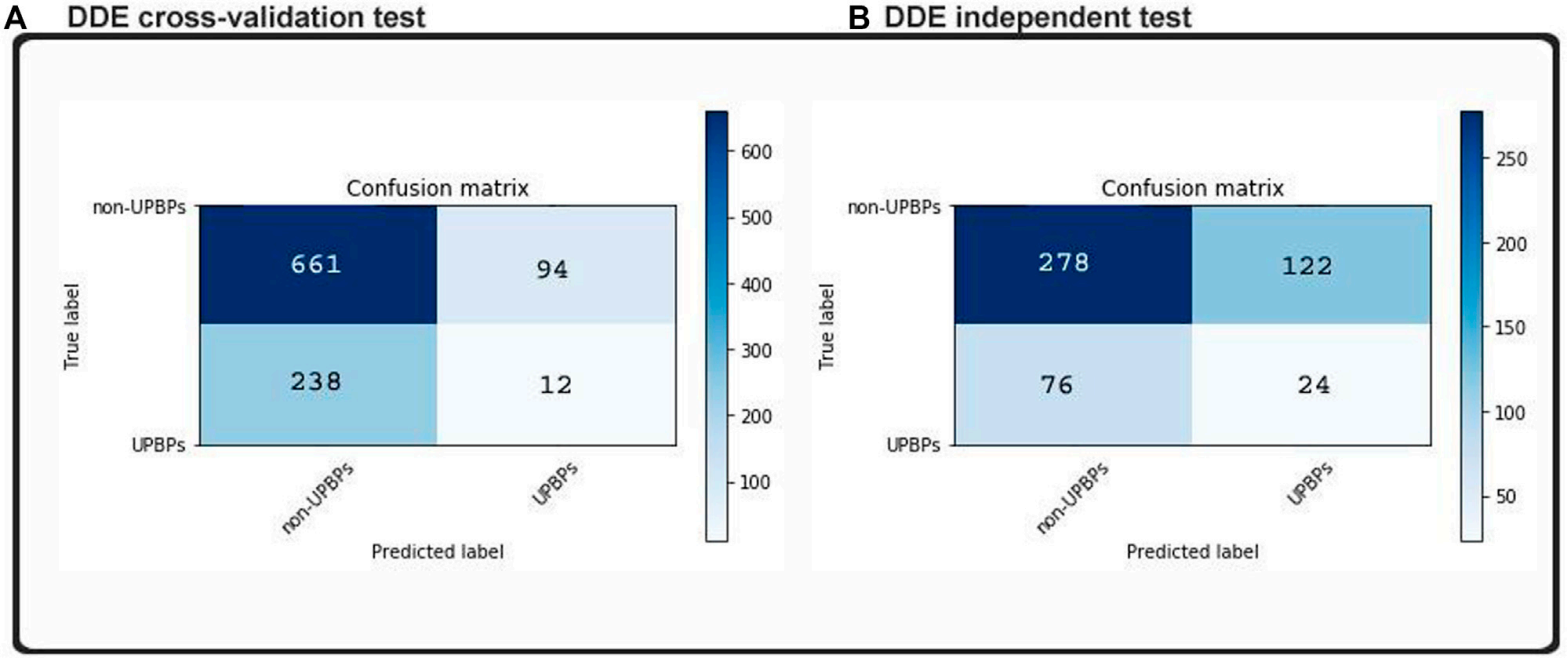
Confusion matrices predicted labels based on **(A)** cross-validation test and **(B)** independent test.

**FIGURE 5:**
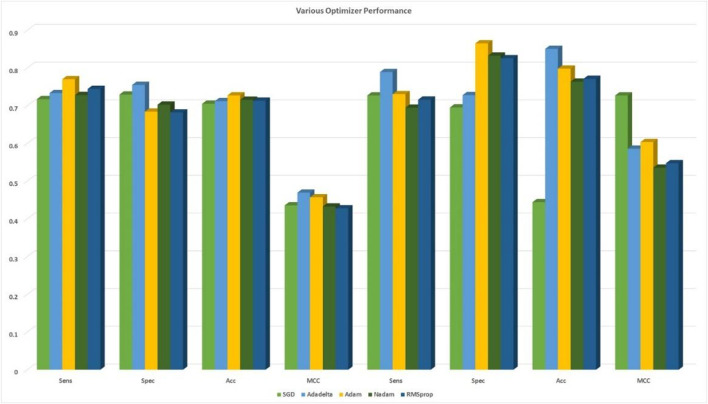
Comparison among the five optimizers based on 10-fold cross-validation with cross-validation and independent sets.

**FIGURE 6:**
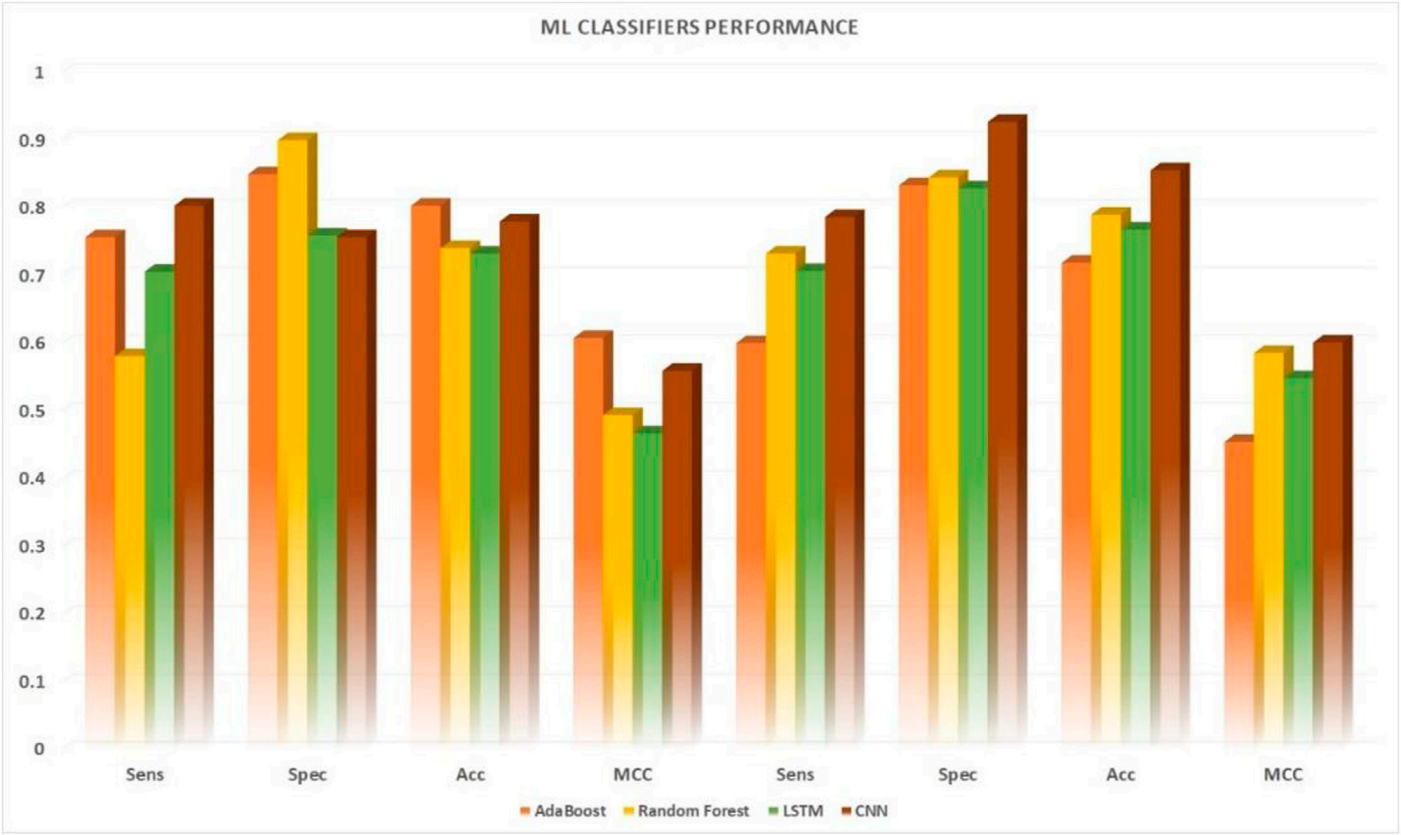
Performance comparison of five machine learning algorithms applied to 10-fold. Cross-validation datasets vs. independent datasets.

Considering that the machine learning method has been perfected to the point of its overfitting, the training dataset, our classifications on new datasets were all highly accurate, but generalizability decreased when we added more examples and instances. This model was subjected to an independent testing, which allowed analyses in the absence of any preexisting information. The independent dataset that we obtained contained 100 UPP and 400 UPP, which were described in the previous section. No cases in the training set could be found in these samples. In addition, two indistinguishable patterns are demonstrated in the matrixes of Figure 4. We found that in our test dataset, the function shown in Figure 6 was equal to the function cross-validation result, which was consistent with our validation result in Figure 5. In our model, percentile accuracy of individual testing was measured (with 80.1% precision, 82% sensitivity, and 69.2% specificity), which set the bar for the other models in our data set and zero points 790 MCC. The accuracy of our model’s cross-validation was lower than that obtained through ordinary procedure, potentially showing that our model was overfit. Dropouts may play a role in further expanding our media, but our CNN initiative was significantly limited by the implementation of CNN-Expand.

### Quantitative techniques on convolutional neural network’s significant function

Extensive methods for training deep learning models were required. Extracted features were specific in the abstract hierarchy, but they were difficult to identify in the models that we use; essential components varied from simple to complex, and they remained a challenge in our CNN model. More valuable knowledge was necessary to provide users an opportunity to learn and information to biologists. When we applied 20 × 20 hybrid feature and matrix feature models to our CNN, we studied the fundamentals of these feature and feature matrix models. We used the F-score to create the list of relevant features that were included in the results. The F-measurement approach aimed to identify the sequences that could be used in UPP and non-UPP. We tried to discover which ones would produce the best results. Here, we showed the entirety of our features and the differences between the two datasets. Figure 5 demonstrates all our features and other features or options. Protein features can be divided into various hidden and prominent classes based on our model. Finally, our model divided amino acids into the prominent and obscure groups. Thus, we may achieve the best classification and conclusion.

### Identification of ubiquitin-proteasome pathway with various optimizer

During content analysis, the optimization of hyperparameters was calculated, but determining the best hyperparameter optimizer for our model was difficult. Most researchers usually aimed to optimize their algorithm performance based on an independent dataset. Several findings from this study warranted further discussion, for example, algorithms for learning. This discrepancy could be due to simplification performance, which was evaluated through cross-validation. The hypothesis that the optimization of the hyperparameters was different from real learning problems, which was considered as an optimization issue, optimized only a loss function. In addition, the learning algorithms could reconstruct their inputs, whereas the optimization of the hyperparameter ensured that the model did not overfit its data by tuning, for example, regularization (Table 5 and Figure 5). The results were considered significant based on the 256 filters used to develop our model for the hidden layer. The neural network were then optimized with several optimizers: RMSprop, Adam, Nadam, SGD, and Adadelta. After each optimization round, a new network was built to make a fair comparison among various optimizers. The model was re-initialized. The performance results are shown in Figure 3. We selected Nadam as our optimizer in creating our final model.

**TABLE 5:** DDE model predicted performance of UPP with different optimizers.

Optimizers	Cross-validation				Independent			
	Sens	Spec	Acc	MCC	Sens	Spec	Acc	MCC
SGD	0.7172	0.7294	0.7049	0.4357	0.7270	0.6951	0.4441	0.7270
Adadelta	0.7334	0.7550	0.7119	0.4693	0.7890	0.7279	0.8505	0.5858
Adam	0.7700	0.6841	0.7271	0.4570	0.7304	0.8650	0.7981	0.6035
Nadam	0.7281	0.7026	0.7154	0.4327	0.6945	0.8324	0.7636	0.5355
RMSprop	0.7444	0.6818	0.7131	0.4277	0.7158	0.8256	0.7709	0.5474

The data were normalized to enhance profound learning architecture by improving and developing new optimization algorithms. The neural networks were challenging, which aimed to reach the optimum through strong training and rapid convergence using algorithms of downward gradient. The basic learning rate of our 2D-CNN was 0.0001, when the Adam battle size optimizer and the maximum number of iterations were used. We used a basic learning rate of 2D-CNN. All layers of CNN were fine-tuned with weights and bias, with a dropout rate of 0.2. The learning rate of the layer was set higher than the previous one. Adam with a learning rate of 0.01 was used for training the 2D-CNN with a decreased kernel weight of 3, 80 epochs, and a batch size of 10. After using five different optimizers based on the DDE feature extraction profile matrix with 2D-CNN models, the creativity of deep learning was evaluated. Afterward, the most appropriate optimizer performance was selected. Finding the best optimizer for the network of 2D-CNN was difficult. A total of five optimal optimizers were selected for performance comparison and classification. Furthermore, RMSprop, Adam, SGD, Nadam, and Adadelta optimizers were compared.

### Performance metirc based on ubiquitin-proteasome pathway with comparable efficiency of shallow neural networks

Various machine learning techniques were used for UPP identification. We utilized four classifications on the basis of five machine learning classifiers (e.g., AdaBoost, Random-Forest, and LSTM). In testing the CNN model, CNN expansions applied a CNN to one dimension of the CNN (2D-CNNs). As shown in Table 6 and Figure 6, we used the highest possible parameters for all experiments; thus, all classifiers would receive equal weighted predicted scores and nearly the same result. We demonstrated that our 2D-CNN had better generalization performance than other machine learning techniques using the same structure. In particular, our separate dataset allowed us to implement the algorithms on the following two-dimensional neural network.

**TABLE 6:** DDE model performance results of identifying UPP with filter numbers.

Filter numbers	Cross-validation				Independent			
	Sens	Spec	Acc	MCC	Sens	Spec	Acc	MCC
AdaBoost	0.7558	0.8488	0.8023	0.6072	0.5996	0.8324	0.7181	0.4539
Random Forest	0.5802	0.8989	0.7396	0.4932	0.7316	0.8439	0.7890	0.5849
LSTM	0.7049	0.7583	0.7316	0.4664	0.7060	0.8281	0.7672	0.5477
CNN	0.8023	0.7558	0.7790	0.5587	0.7857	0.9259	0.8545	0.6005

### Ubiquitin-proteasome pathway identification by using ROC- a comparative performance

**FIGURE 7:**
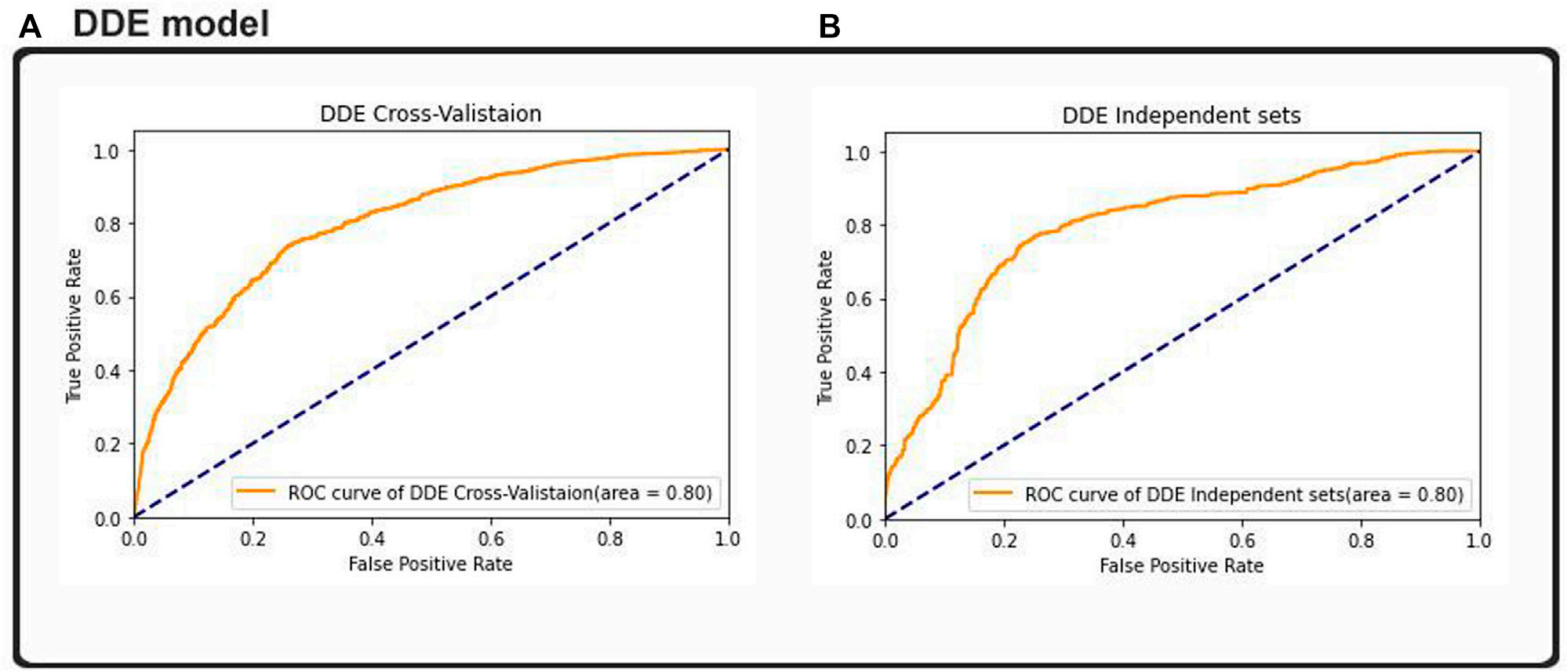
ROC–AUC calculation based on the **(A)** cross-validation test and **(B)** independent test.

Result analysis measured the effectiveness of the performance of the binary classification problem, which was comparable to other studies measuring other binary classification techniques. Almost all of the machine learning classifiers used provided good data to support our assumptions. The researchers used the ROC curve and other metrics, such as algorithm’s accuracy and the metric of the extent of confusion, to plot a graphical representation of their predictive output. The results from Figure 7 were plotted using the ROC and AUC techniques to explore how the output of the 2D CNNs was affected by different classifications. The multilink ROC curve plot was expanded to show the parts of the up-and-down double down CURVE. The results indicated that our deep neural network architecture can perform with the binary method, but additional multiclassification was required to explore this result further. Thus, our 2DC model showed the best generalization and little-to-no overfitting, and asymtote tolerance was maintained by cross-validation. Moreover, cross-validation and non-overfitting greatly limited generalization using twofold and threefold cross model results. Therefore, the zero overfitting 2DC result was aided by cross-validation, but the other results avoided cross model overfitting. Furthermore, the results with the same datapoints demonstrated that the DDE model validation datasets had an area under the ROC curve (AUC) of 0.80%, whereas the dataset comparison showed that the ACU of the same datasets was 0.80%. However, our findings and the ROC found that the DDE composition (ROC has been calculated at 0.80) was consistent with those of previously published research studies with 10-fold cross-validation results; thus, the validity of our findings was 0.80% with independent sets. This finding indicated the efficacy of the procedure in accomplishing this objective. In testing the overall performance of the 2D-CNN UPP, different datasets were utilized, and the results of the 2D-CNN UPP output were found. In addition, the results of the three machine learning classifiers were provided for comparison, all of which had a ROC AUC score of 0.80% with regard to AdaBoost. This classifier used random-forest as its ROC-AUC classifier, and an LSTM model used ROC AUC as its ROC-AUC classifier.

## Case study

### Ubiquitin protein-pathway association

In a human reference standard cDNAs, including Arabidopsis FUS6, the signal transduction pathway was inhibited *via* G-protein and kinase activator proteins (Chae et al., 2013). A unique pathway, known as the arginosidase pathway, inhibited melanoma tumor by tumor-specific T cells. The UniProt Knowledgebase records Q13227 (GPS2 HUMAN) indicated an association among pathways, such as Q13227, which had a unique identifier. Protein Q13227 inhibited UBE2N/Ubc13. When the mitochondrial membrane was depolarized, the role shifted from the mitochondria to the nucleus and cleaned out the mitochondria-encoded expression (Palvimo, 2007). Considering that GPS2 was expressed in the mitochondrial and nuclear regions, it was considered as a mediator of retrograde and transcription factor that promoted tumorigenesis. These results showed an additional mitochondrial transcriptional regulation and a nuclear mitochondrial pathway and indicated that an important component of the mammalian stress response to mitochondrial damage was guided by a retrograde GAP2 signal.

**FIGURE 8:**
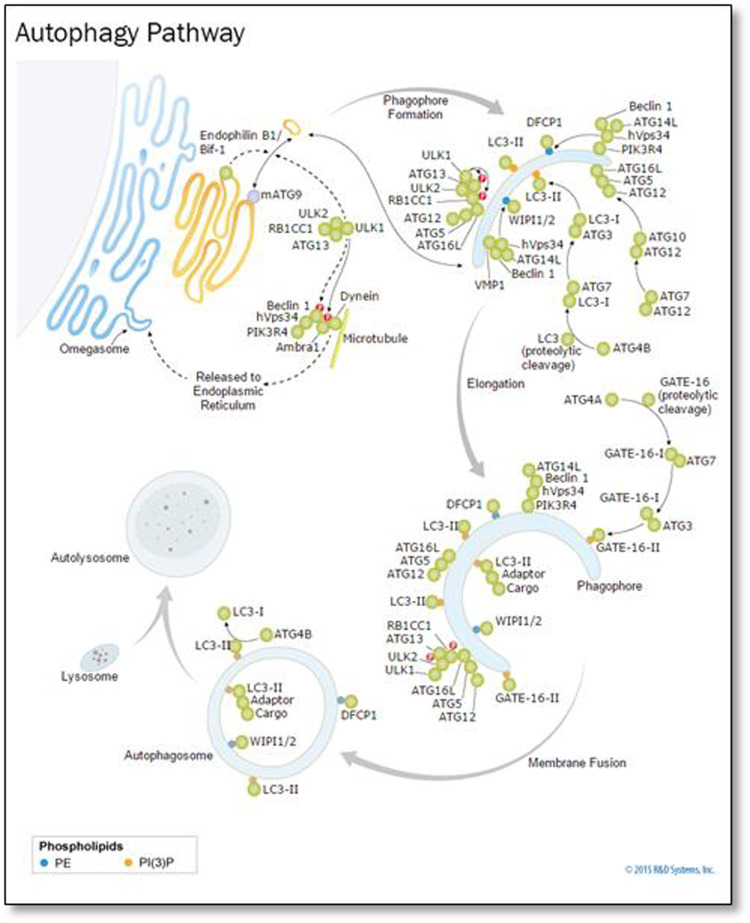
Ubiquitin, related modifiers, and pathways (R&D Systems Europe, Ltd).

In the cytoplasm and nucleus, ubiquitin was conserved in the form of a 78-residue complex. Ubiquitination is a process in which Ubiquinone is linked to proteins as shown Figure 8. This process has an impact on protein localization, protein interaction, and protein stability (Ciechanover, 1998). Recent studies have found that ubiquitination is involved in cellular regulation, if not all, in a number of diseases and pathological conditions.

### Ubiquitin-proteasome pathway

**FIGURE 9:**
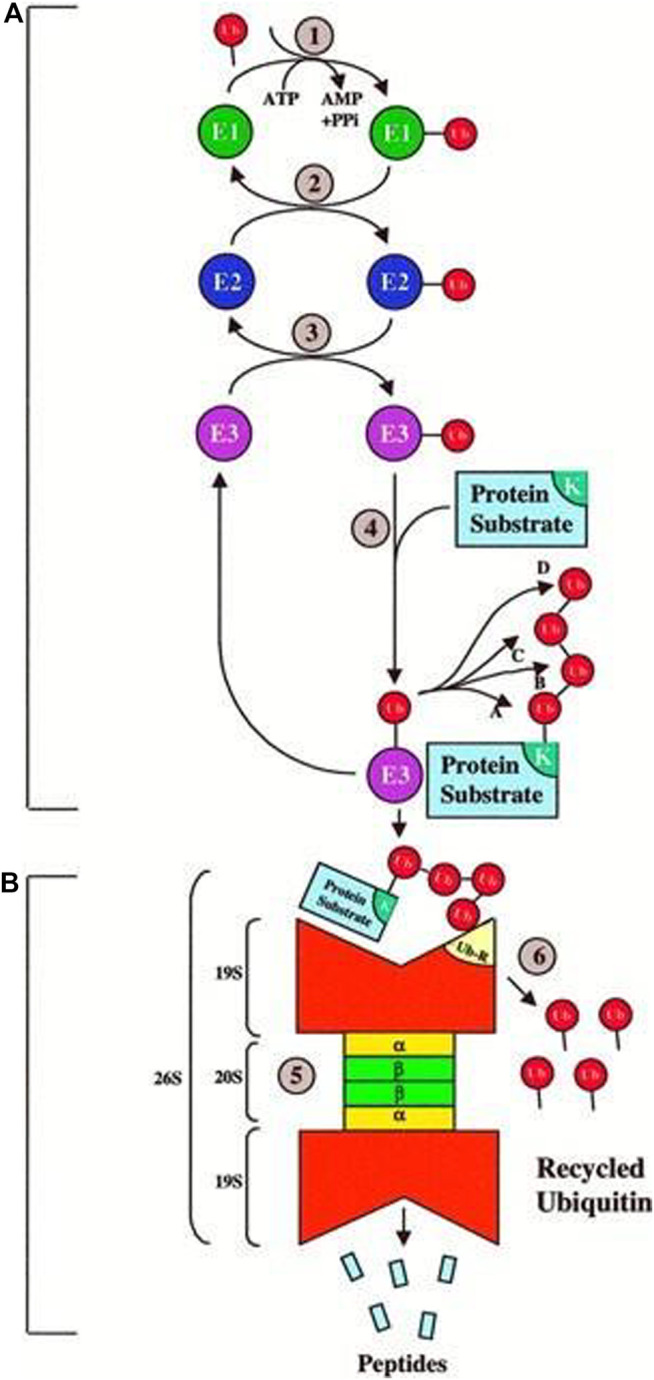
Ubiquitin–proteasome pathway (Ciechanover, 1998)

Previous research has largely overlooked the challenges associated with UPP. The activities of different proteins can be controlled in the UPP. The UPP is the main negative mechanism of regulating the proteins. Ubiquitin gives the 26S proteasome a recognition signal and protein modified with Ubiquitin, and the transportation factor of the 26S proteasome somewhere committed receptors start its degradation. This complex biochemical machinery has been ingeniously divided into two distinct steps as shown Figure 9.

Step A: is a particular process of recognition, which uses the Ubiquitin pairing flow and an integrated method for different combinations. In particular, three sets of enzymes shuttle Ubiquitin (e.g., E1, E2, and E3) and ultimately connect Ubiquitin with the protein substratum. Various sequences of E2 or E3 or both recognize the unique degradation signal of each substrate, giving the various protein substrates exquisite ubiquity.

Step B: is a process of indiscriminate destruction through the proteolytic proteasome core, degrading the tagged substrate by the 26S proteasome. This indiscriminate proteolytic step gives the signaling pathway direction, that is, when a protein has been committed to degradation, no return occurs to prevent the interference of biological processes with partially degraded proteins. Finally, the 26S proteasome recognizes the majority of ubiquitinated proteins, and it is unfolded and filleted in an ATP-dependent manner into the 20S proteolytic chamber.

### Procedure and control of the ubiquitin proteasome pathway

Research on ubiquitin as a regulatory protein of 9 kDa, which binds on the proteins in the substrate to create a post-translational change, is limited. The long poly-ubiquitin chains are intended for 26S proteasome degradation. This process discriminates tagging and degradation of specific intracellular proteins with various combinations of E2 and E3 enzymes. By contrast, one highly conserved E1 family is identified.

Few studies have investigated that the effect of Ubc13 protein forms part of the enzyme families conjugating Ub (E2 enzyme) and receiving Ub (E1 enzyme). TNFα induces poly-ubiquitinated receptor-interacting protein kinase (RIP) and NEMO (NF-α essential modulator) RIP-associated inhibitors of the IKKβ and IKKα nuclear factor kinase complex. The ubiquitination of I5-0B results in a separation of the ubiquitinated IμB from NF-ŚB, and it is directed to the 26S proteasome, that is, supplemented by valosin-containing peptide. NF-ŢB is released into the nucleus to activate the transcription of target genes.

### Ubiquitin-proteasome pathway based on pathologies

In recognition of the key importance of UPP in biology, the Nobel Prize for Chemistry was presented in 2004 to Avram Hershko, Aarán Ciechanover, and Irwin Rose. UPP plays an important role in regulating various processes in cells, which affect DNA transcription, cell cycle, inflammation, biogenesis of ribosome, and soon. Deficiencies in different UPP components have led to a variety of human diseases, including cancer and neurodegenerative diseases, making this pathway a potential therapeutic approach.

### Research of the ubiquitin-proteasome pathway

The UPP must be given considerable research attention to uncover new areas of significance for ubiquitin as a degradation target. Evidence of enzyme-linked system of protein breakdown, namely, the lysosomal system (lysosin and UPP), in eukaryotic cells is limited. Bacteria were expected to play a significant role in the majority of the protein intracellular degradation. Considering that the half-life of the proteins differed between lysosomal proteolysis and the amount of time that proteins were degraded during exposure to physiological stimuli, different classes of proteins had different amounts after examination of the samples from the lysosome. In addition, the removal of internal lysosin proteins used ATP, which was at odds with what was previously thought about lysosin protein degradation. In the 1980s, the ubiquitin-proteome (proteolytic pathway) system differed from the lysosomal (karyophero) systems in eukaryotic cells, which caused the development of the ubiquitin-proteasome (translocation) pathway to be recognized.

The search for intracellular protein degradation was originally a quest for proteolysis to be reconstituted in a non-dependent system, but later began to concentrate on ATP. One researcher, Avram Hersh, utilized the reticulocyte lysate system as the basis for proteolysis research on proteolysis, which was created by Etlinger and Goldberg. ATP was elucidated using reticulocyte lysates and lyzymes, such as globin, and Avram Hersh’s students Aaron Ciechan, Irwin Rose, and Aaron Rose developed a conceptual reticle to define ATP. This unique component appeared to be involved in ATPase activity because it was found to be linked to a heat-stable factor, namely, APF1. According to Wilkinson et al., APF-1 was later discovered to be ubiquitin by other researchers. The ground-breaking research by Hershko, Ceich, Rose, and Gane was honored by the award of a Nobel Prize in chemistry in 2004.

**FIGURE 10:**
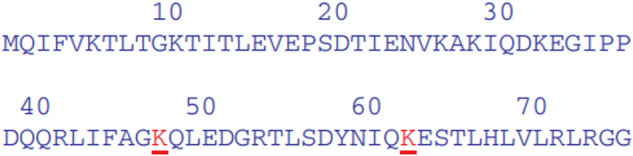
Ubiquitin protein pathway amino acid sequence.

Ubiquitin has an amino acid sequence. The entire sequence of ubiquitin can be visualized from a set of amino acid letters. In addition to the key lysine (K), the 48th and 63rd position of each amino acid is typed as “expanded.” These symbols are given in red. The vast majority of the work in this area has focused on ubiquitin as a small peptide of 76 amino acids (Figure 10). Prior to its identification as thymidine kinase, a component of the enzyme has been discovered (APF1-10). Given the evidence available at the time, APF1-10 was assumed to be an elemental property of all living cells. Since ubiquitin is ubiquitous among all cells, it is well conserved, making it an important protein. The three amino acid residue differences between yeast and human ubiquitin that separate yeast and human ubiquitin are 100% identical. Aplysian and human oligopeptophillin are another human proteolin, which are slightly different from those used to fight influenza and herpes. Fibrin is found in all eukaryotes, but it does not occur in the protista. The polyubiquitin gene encodes several ubiquitins linked without any introns. The number of ubiquitin coding repeats is around 5 or 6. Polyubiquitins (also called polyubiquitin genes) have 52 overlapping genes, tandemly coding polyproteins in species such as Trypanosoma cruzi. Several genes, which have an ubiquitin-coding sequence, are fused to sequences that carry out small ribosomal subunit genes on a translocator protein. The basic ubiquitin one is derived from adenine, and ubiquinol A is found in the compound ubiquinone metabolic pathway (monoubiquitin).

## Protein-protein interaction of ubiquitin protein pathway genes

**FIGURE 11:**
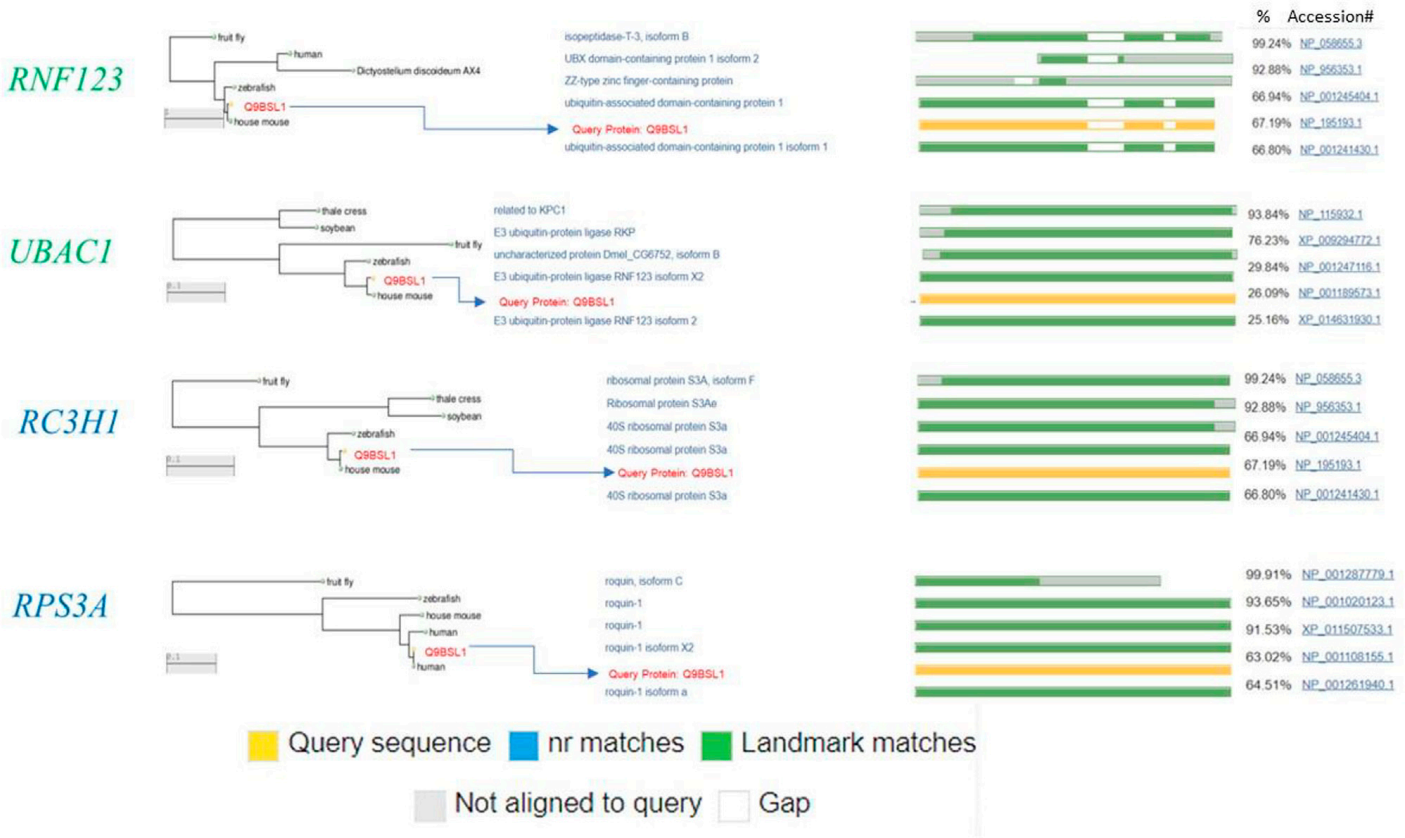
Conservational Analysis of RNF123, UBAC1, RC3H1, and RPS3A genes. Multiple protein sequence alignment and Phylogenetic tree was performed by SmartBLAST. Parentheses refers to the percent sequence of identity of the reference sequence.

Further we analyzed the protein-protein interactions of all identified genes, as listed in Table 7, and found that the RC3H1 protein is interlinked with the RPS3A protein. Based on STRING experiments (stringdb) and scores (0.876 and 0.998), the RNF123 protein is highly interlinked with the UBAC1 protein (Figure 11). The STRING analysis results showed that all interacting proteins play an essential role in disease. Additionally, it can be claimed that any change in the interaction of these proteins can cause a change in its associated pathway, leading to the onset of the disease.

**TABLE 7:** Providing ubiquitin protein-pathway association evidence.

Entry	Entry name	Protein name	Gene name	Organism	Pathway Ids
Q13227	GPS2_HUMAN	G protein pathway suppressor 2	GPS2	*Homo sapiens*	R-HSA-1989781
Q13098	CSN1_HUMAN	COP9 signalosome complex subunit 1	GPS1, COPS1, CSN1	*Homo sapiens*	R-HSA-5697010
Q9Y618	NCOR2_HUMAN	Nuclear receptor corepressor 2	NCOR2	*Homo sapiens*	R-HSA-383280
Q9NQS5	GPR84_HUMAN	G-protein coupled receptor 84	GPR84, EX33	*Homo sapiens*	R-HSA-418555
C9JFE4	C9JFE4_HUMAN	COP9 signalosome complex subunit 1	GPS1,1987516	*Homo sapiens*	PTHR14145:SF2
A8K070	A8K070_HUMAN	COP9 signalosome complex subunit 1	GPS1	*Homo sapiens*	PTHR14145:SF2
I3L3Y9	I3L3Y9_HUMAN	G protein pathway suppressor 2	GPS2	*Homo sapiens*	PTHR22654
I3L1H4	I3L1H4_HUMAN	G protein pathway suppressor 2	GPS2	*Homo sapiens*	PTHR22654
I3L242	I3L242_HUMAN	G protein pathway suppressor 2	GPS2	*Homo sapiens*	PTHR22654
I3L4X7	I3L4X7_HUMAN	G protein pathway suppressor 2	GPS2	*Homo sapiens*	PTHR22654

**FIGURE 12:**
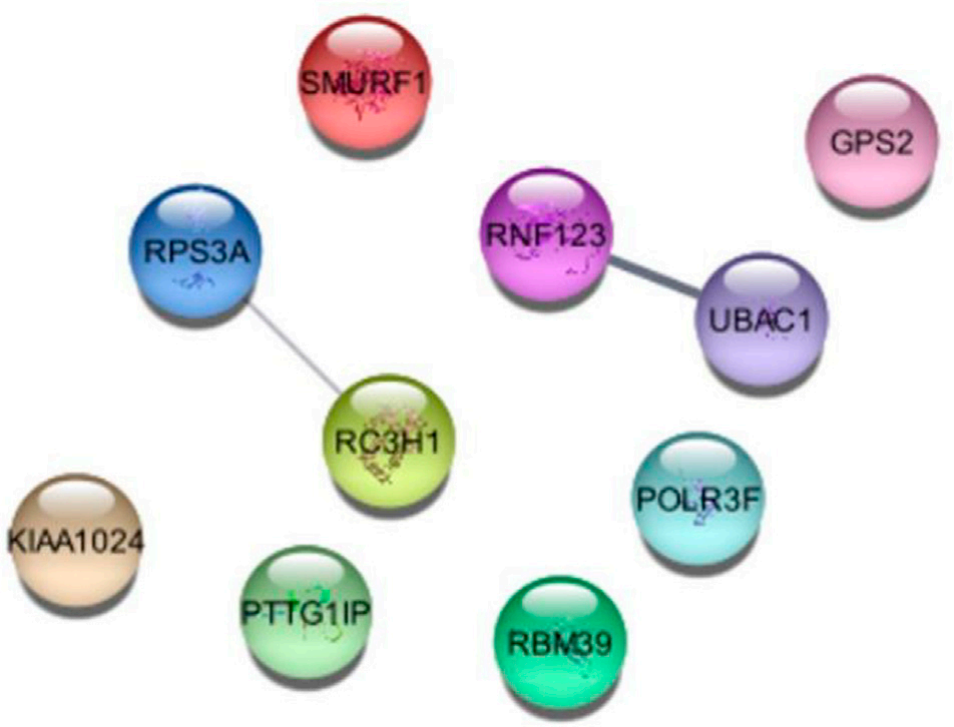
Protein-Protein interaction of identified genes of ubiquitin protein-pathway.

To consolidate the conservational analyses of protein-protein associated genes (RC3H1, RPS3A, RNF123, and UBAC1), we analyzed multiple-sequence-alignment and phylogenetic tree that illustrated a sequence similarity, suggesting that our identified ubiquitin protein pathway-related genes are conserved among different species as shown in Figure 12.

## Future direction

Based on these studies, considerable research was conducted to design effective and significant education models for UPP/non-UPP. Several, ubiquitin-pathway-specific proteins are bound to disease and toxins and associated with proteins (e.g., genes, drugs, and enzymes). At present, some ubiquitin pathways are related (directly or likely) to disease. Integration is important to broaden the scope of research and determine the importance of the ubiquitin-pathway compounds. Further research is needed to investigate the interaction of ubiquitin protein pathway-related genes.

## Discussion

One ubiquitin pathway becomes covalently linked to other molecules or polymers in a monomeric or polymeric form. Enzyme E1 (ubiquitin activating) requires an ATP-dependent enzymatic process, namely, E2 (ubiquitin conjugating) and E3 (ubiquitin ligating), to bind ubiquitin to the lysine moieties of proteins. An E2–E3 complex is formed by binding to a substrate, which may be the key to the complex recognition and formation of the conjugation cascade. This hydrophobic amino acid C-terminal aliphatic linker of ubiquitin is essential in many ubiquitin-mediated reactions. Different linkages in UBIDE are bound to certain lysine (K6, K11, K27, K29, K33, and K48). This finding implies that specific ubiquitin-pathway enzymes can also remove the covalent bond between ubiquitin binding and a target protein (UBs). Recent studies have found that UBs serve as dynamic enzymes that bind substrates together with multiple proteins and lead to UBs during substrate activation. The separate expression of monobit and poly ubiquitin has provided an array of UB activities necessary to handle the increasingly diverse substrates. The ubiquitin proteome system is also underlined by a growing body of evidence, which demonstrates that many UBs are found in ubiquitin ligase complexes, thereby ensuring that they control the ubiquitin ligase and level of abundance of the substrate. Another class of UBs and pathways with their functions in the cellular context are listed below.

## Conclusion

We developed novel 2D-CNNs for UPP quality prediction. An entire protein model of subjective length, rather than having a fixed-size window, was used to produce features for each residue in the feature pipeline. This process gives us the ability to access structural information when generating the features for the protein structure. We have designed a new training pipeline that integrates the 2D-CNN. The results confirmed that 2D-CNN classifiers can be used for protein evaluation. Advanced CNN architectures will be able to advance protein modelling in the near future. Despite ubiquitin being the most well-modeled post-translational modifier, a steadily increasing group of ubiquitin-like proteins (BLs) working in a parallel is observed, but in a separate pathway. The additional alternative operators include SUMO, NED, FAT, ISG, and UFM. These related molecules perform novel activities and diverse functions, and they have novel influences in biology. In addition, the various conjugation proteins may bind to ubiquitin, which can be modified at the same lysine residue. Moreover, SUMO modification often has the effect of destabilizing substrates. In general, proteases are not involved in proteasomal degradation, but they perform diverse functions. UBL attachment might change the conformational equilibrium, ligand binding affinities, and protein localization. UBLs conjugated with the enzymatic mechanisms are structurally similar to ubiquitin, activated, and released from conjugates, although their proteolytic processing may differ alternatively. UBLs are exposed with LRGG object-oriented code-translated extensions, which turn the objects into terminals. Carriers to targets conjugate the free E1 (activated), E2 (semi-activated), and E3 (hydrolyzed). These ubiquitin pathways can be deactivated using deoxyribonuclease enzymes, which have the same manner of action as deacidifying enzymes. Thus, the anticipated outcomes reveal that our research will provide remarkable contribution in large-scale UPPs identification and research academia (Le and Nguyen, 2019).

## Data Availability

The original contributions presented in the study are included in the article/supplementary material, further inquiries can be directed to the corresponding authors.
